# In Vitro Antimicrobial and Antioxidant Activities of *Lactobacillus coryniformis* BCH-4 Bioactive Compounds and Determination of their Bioprotective Effects on Nutritional Components of Maize (*Zea mays L*.)

**DOI:** 10.3390/molecules25204685

**Published:** 2020-10-14

**Authors:** Mahwish Salman, Anam Tariq, Anam Ijaz, Shazia Naheed, Abeer Hashem, Elsayed Fathi Abd_Allah, Mona H. Soliman, Muhammad Rizwan Javed

**Affiliations:** 1 Department of Biochemistry, Government College University Faisalabad (GCUF), Allama Iqbal Road, Faisalabad 38000, Pakistan; mahwishsalman@gcuf.edu.pk (M.S.); anam.tariq48@yahoo.com (A.T.); 2 Department of Bioinformatics and Biotechnology, Government College University Faisalabad (GCUF), Allama Iqbal Road, Faisalabad 38000, Pakistan; anumijaz1246@gmail.com; 3 Department of Chemistry, Government College University Faisalabad (GCUF), Allama Iqbal Road, Faisalabad 38000, Pakistan; shazia.gcu@hotmail.com; 4 Botany and Microbiology Department, College of Science, King Saud University, P.O. Box. 2460, Riyadh 11451, Saudi Arabia; habeer@ksu.edu.sa; 5 Mycology and Plant Disease Survey Department, Plant Pathology Research Institute, ARC, Giza 12511, Egypt; 6 Plant Production Department, College of Food and Agricultural Sciences, King Saud University, P.O. Box. 2460, Riyadh 11451, Saudi Arabia; eabdallah@ksu.edu.sa; 7 Botany and Microbiology Department, Faculty of Science, Cairo University, Giza 12613, Egypt; hmona@sci.cu.edu.eg

**Keywords:** *Lactobacillus coryniformis*, antimicrobial compounds, maize (*Zea mays* L.), HPLC, FTIR

## Abstract

Lactic acid bacteria (LAB) can synthesize antimicrobial compounds (AMCs) with nutritional and bioprotective properties in crops and food products. In the current study, AMCs of *Lactobacillus coryniformis* BCH-4 were evaluated to control fungal spoilage in maize grains. On maize grains treated with 75%–100% (*v*/*v*) concentrated AMCs, no fungal growth was observed even after 72 h of *Aspergillus flavus* inoculation. Proximate analysis of treatments A1 (raw grains), A2 (*A. flavus* inoculated grains) and A3 (*A. flavus* + AMCs inoculated grains) revealed that moisture was significantly (*p* ≤ 0.05) high in A2 than A3 and A1. Meanwhile, protein, fat, fiber and ash contents were significantly decreased in A2 compared to A1 and A3. Moreover, β-carotene contents were not statistically different between A1 and A3, while in A2 it was significantly decreased. HPLC analysis revealed the presence of 2-oxopropanoic acid, 2-hydroxypropane-1,2,3-tricarboxylic acid, 2-hydroxybutanedioic acid, 2-hydroxypropanoic acid, propanedioic acid and butanedioic acid, which also showed antifungal activity against *Aspergillus flavus*. FTIR spectroscopy revealed the presence of hydroxyl, carbonyl and ester-groups along with organic and fatty acids, thereby indicating their participation in inhibitory action. Furthermore, the AMCs were found to be a good alternative to chemical preservatives, thereby not only preserving the nutritive qualities but increasing the shelf life as well.

## 1. Introduction

Deterrence of food from pathogenic microorganisms has always been a major task. The harmful products of these microorganisms endanger human as well as animal health [1]. Moreover, the high yield potential of plant species and crop cultivars become increasingly prone to diseases because of many pathogenic microorganisms that are major causative agents of spoilage, including bacteria, viruses, yeast and fungi [2]. Among them, pathogenic fungi (e.g., *Blumeria graminis*, *Aspergillus flavus*, *Botrytis cinerea*) are a major challenge to crops as they produce various toxic products, e.g., aflatoxins, linoleic acid, botcinic acid and their derivatives, which ultimately damage plants and spoil their products [3,4,5]. Fungi are etiological agents in many crop diseases and account for substantial losses at both pre- and post-harvest stages of crop production [6]. The financial losses due to post-harvest spoilage of cereal crops often appear in the form of low yield [2].

The production of cereal grains varies yearly; these grains should be stored from the year of over-production to the year of under-production. These stored grains can lose their quality because post-harvested cereal grains are influenced by various biotic and abiotic factors [7]. Among them, moisture is the most important factor regarding seed viability during storage. The moisture content above 13% in starchy cereal grains, namely wheat, rice and maize, leads to the invasion of storage fungi (predominant fungal genera include *Aspergillus, Alternaria*, *Cladosporium*, *Fusarium*, *Mucor*, *Rhizopus* and *Penicillium*) as the storage time and temperature increases [8,9].

Maize (*Zea mays* L.) ranks the 3rd most important cereal grain worldwide, after wheat and rice. It is a multipurpose crop being used as a staple food, for feed and biofuel production. The demand for maize in the developing countries is expected to double by 2050 [10]. The fungal genera that commonly infect maize are *Fusarium*, *Penicillium*, *Rhizopus*, *Aspergillus* and *Tilletia*. Fungal infection results in discoloration, lower nutritional values, and reduced quality and yield. Moreover, these pathogens also cause maize rot, kernel discoloration, loss of viability, mycotoxin contamination and subsequent seedling blights [11,12]. Chemical preservatives such as benzoates, nitrates and sorbates have been extensively used for the protection of post-harvested cereal grains; however, the use of such preservatives ultimately affects the nutritional qualities of grains [13,14]. In order to look for alternatives, beneficial microorganisms and their end products such as lactic acid bacteria, e.g., *Lactobacillus plantarum* [1], *L. acidophilus*, *L. delbrueckii* [15] and *Lactococcus lactis* [16] have been identified by researchers to overcome a high susceptibility to spoilage and to get good quality, chemical preservative-free food as demanded by the consumers [17].

Lactic acid bacteria (LAB) have been widely used for bioprotection purposes to enhance the shelf life of food [18]. LAB being used as biopreservative organisms are of prime significance; they have been employed for years as a starter culture in food industries. They increase the shelf life of food because of their inhibitory actions on food borne pathogenic microorganisms [19]. They are present normally in gut microbiota and in several dairy products, such as fermented milk and cheese, and play an important role in human health following oral administration [20]. These bacteria produce a variety of bioactive compounds including organic acids, phenyl lactic acid, bacteriocins, cyclic dipeptides, reuterin and hydrogen peroxide [21,22,23].

*Lactobacillus coryniformis* is a facultative anaerobic species of lactic acid-producing bacteria. This strain produces broad spectrum antimicrobial compounds (AMCs) such as lactic acid, acetic acid, hydrogen peroxide, bacteriocins and reuterin (3-hydroxypropionaldehyde, 3-HPA) [24]. It has shown inhibitory activity against molds *Aspergillus fumigatus*, *A. nidulans*, *Penicillium roqueforti*, *Mucor hiemalis*, *Talaromyces flavus*, *Fusarium poae*, *F. graminearum*, *F. culmorum*, and *F. sporotrichoides* [25]. In the present study, the focus was on the extraction of bioactive compounds from *L. coryniformis* BCH-4 to elucidate in vitro antimicrobial and antioxidant activities. The potential of AMCs for bioprotection of maize grains to overcome post-harvest loss has also been evaluated. Furthermore, high-performance liquid chromatography (HPLC) analysis was carried out to identify and determine the concentration of organic acids in AMC extracts. The functional groups were determined by Fourier Transform Infrared (FTIR) Spectroscopy. This is the first report of its kind, as no work has been reported earlier for the bioprotection of post-harvested maize by using bioactive antimicrobial compounds of *L. coryniformis*.

## 2. Results

### 2.1. Potential of Antimicrobial Compounds (AMCs)

**Figure 1:**
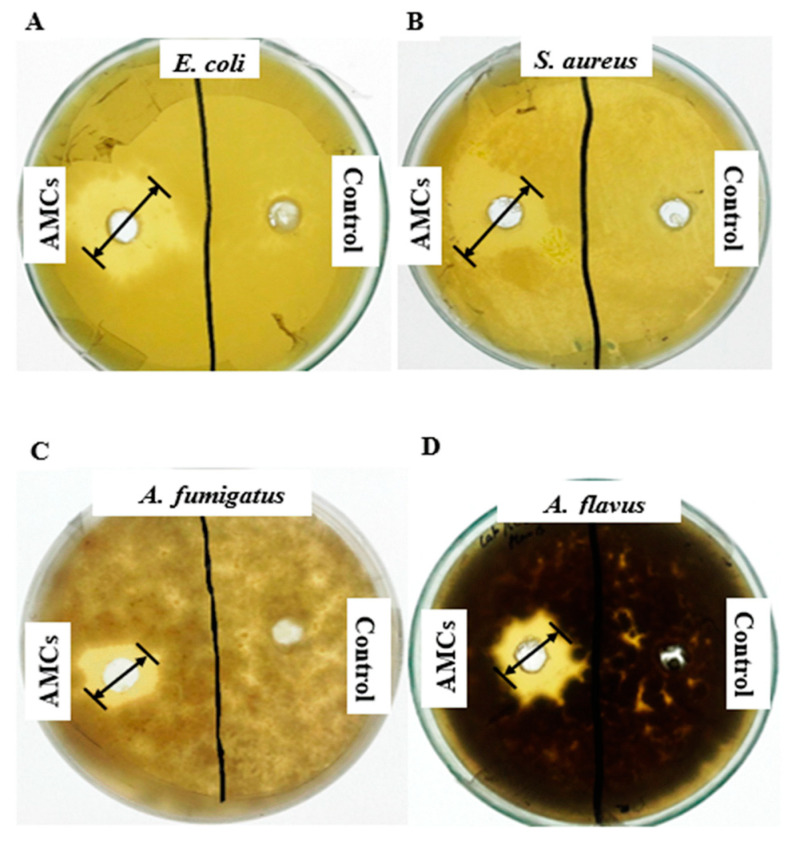
Antimicrobial activity of *L. coryniformis* BCH-4 ethyl acetate extracted antimicrobial compounds (AMCs) after 16 h of incubation for antibacterial and 48 h of incubation for antifungal activity. (**A**) *E. coli* (Gram-negative shiga toxin producing bacterial strain); (**B**) *S. aureus* (Gram-positive multi-drug resistant bacterial strain); (**C**) *A. fumigatus* (Saprotrophic filamentous fungus) (**D**) *A. flavus* (Aflatoxigenic filamentous fungus). de Man, Rogosa and Sharpe (MRS) broth was used as a negative control and the well diameter was 8 mm.

The low molecular weight secondary antimicrobial compounds (AMCs) of *L. coryniformis* BCH-4 showed strong antimicrobial activity against tested pathogenic fungal and bacterial species. Clear zones of inhibition appeared (Figure 1) with mean inhibition (*n* = 3) of 15.00 ± 1.00, 14.66 ± 1.52, 9.00 ± 1.00 and 12.33 ± 0.57 mm, respectively, against pathogenic strains of *Escherichia coli*, *Staphylococcus aureus*, *Aspergillus fumigatus*, and *Aspergillus flavus*. No activity was observed in negative controls against these test strains.

### 2.2. Antioxidant Activity (DPPH Scavenging Activity)

DPPH (2,2-Diphenyl-1-picrylhydrazyl) radical scavenging activity of AMCs produced by *L. coryniformis* BCH-4 showed potent activity in a concentration-dependent manner. Mean inhibition (± SD%) of DPPH activity at 25, 50 and 100 (μL/mL) was 30.28% ± 1.03%, 48.58% ± 0.50% and 61.66% ± 0.55%, respectively (n = 3). The results show that the highest concentration of AMCs tested contributes maximum scavenging of DPPH.

### 2.3. Comparative Antifungal Analysis of AMCs with Commercial Preservatives

**Figure 2:**
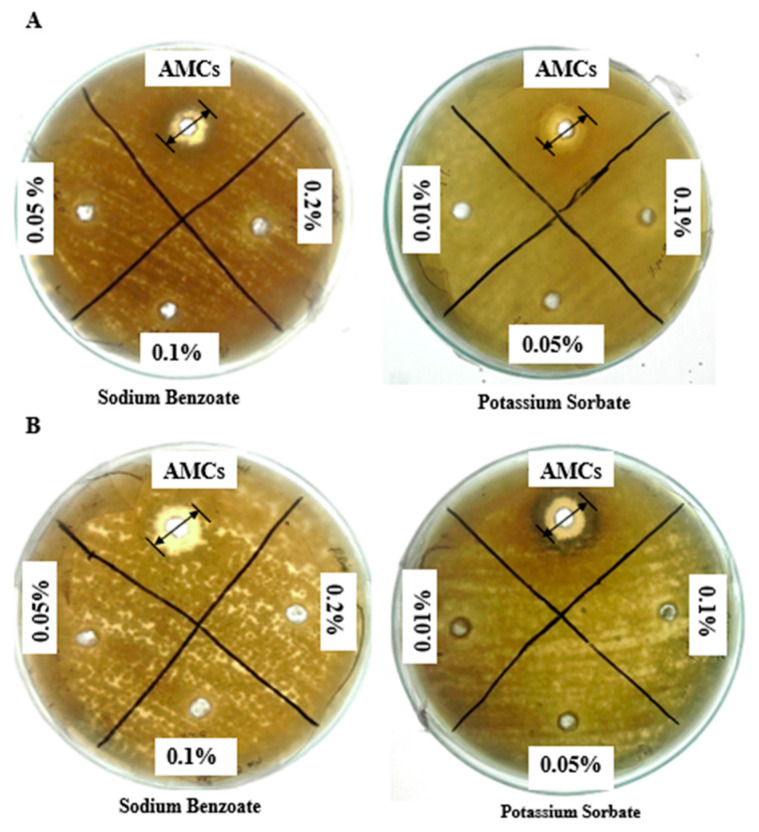
Inhibition zones of antifungal potential of *L. coryniformis* BCH-4 ethyl acetate extracted AMCs in comparison to commercial preservatives (sodium benzoate and potassium sorbate) against (**A**) *A. fumigatus* and (**B**) *A. flavus* at 30 °C after 48 h of incubation. Well diameter was 8 mm.

A comparative antifungal study of extracted AMCs with FDA (Food and Drug Administration)-approved concentrations of preservatives (sodium benzoate and potassium sorbate) was carried out (Figure 2A,B). The AMC concentrations being studied showed maximum growth inhibition of *A. fumigatus* (8.98 ± 1.00 mm) and *A. flavus* (12.00 ± 0.56 mm) in comparison to the FDA-approved highest concentrations of sodium benzoate (0.2% *w*/*v*; 1.44 ± 0.57 mm) and potassium sorbate (0.1% *w*/*v*; ~0.00 mm) after 48 h of incubation.

### 2.4. Efficiency of AMCs for Bioprotection of Maize

**Figure 3:**
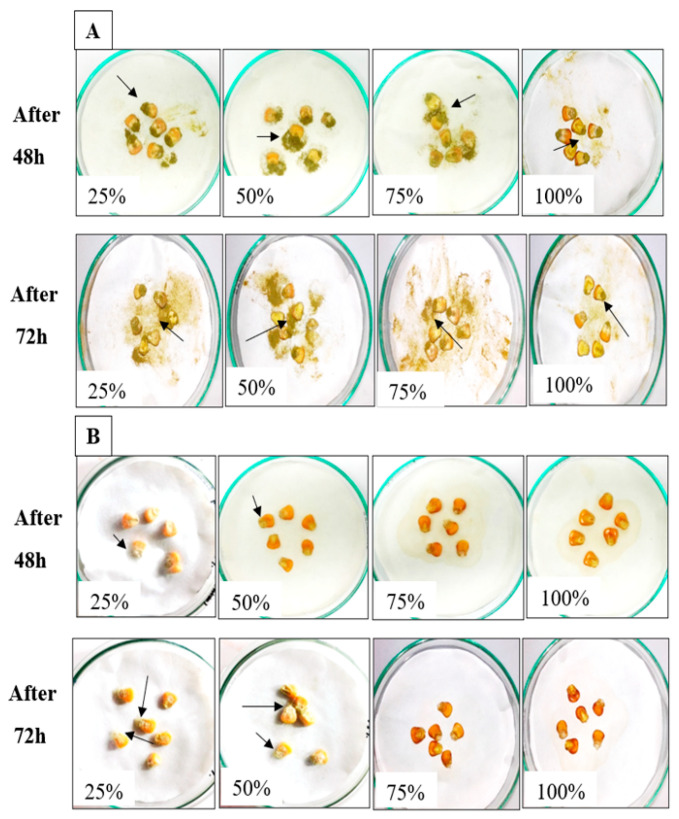
*A. flavus* growth on raw maize grains soaked with (**A**) MRS broth [25%, 50%, 75% and 100% (*v*/*v*)] as control and (**B**) *L. coryniformis* BCH-4 AMCs [25%, 50%, 75% and 100% (*v*/*v*) AMCs] treated maize grains after 48 and 72 h of incubation at 30 °C. Arrows indicate the white mycelia and/or green spores of *A. flavus.*

AMCs of *L. coryniformis* BCH-4 showed highly potent antimicrobial activity against pathogenic strains, therefore maize grains were used as a food model that is considered as a staple food in many countries and primarily affected by *A. flavus* during the storage period. In maize grains soaked with de Man, Rogosa and Sharpe (MRS) broth (control), dark green spores were observed after 48 h of incubation (Figure 3A). Meanwhile, few white mycelia of *A. flavus* were observed in 25% (*v*/*v*) and 50% (*v*/*v*) AMC concentration-treated maize, whereas no growth was observed in 75% (*v*/*v*) and 100% (*v*/*v*) treated concentrations (Figure 3B), even after 72 h.

### 2.5. Proximate Analysis and β-Carotene Determination of Untreated and Treated Maize Grains

The proximate analysis of treatments A1 (raw maize grains), A2 (*A. flavus* inoculated maize grains) and A3 (*A. flavus* + AMCs inoculated maize grains) are summarized in Table 1. The moisture content of A2 was higher than A1 and A3, but there was no significant difference (*p* > 0.05) between the moisture contents of A1 and A3. Meanwhile, protein, fat, fiber and ash contents were significantly (*p* ≤ 0.05) decreased in A2 compared to in A1 and A3, as summarized in Table 1. Moreover, β-carotene contents in A1 and A3 were not statistically different, while a significant decrease has been observed in A2. Consequently, AMCs treatment produced by *L. coryniformis* BCH-4 significantly improved the nutritional values of maize grains and also protected them from *A. flavus* infestation.

### 2.6. HPLC Analysis and Antifungal Potential of Low Molecular Weight Organic Acids

**Figure 4:**
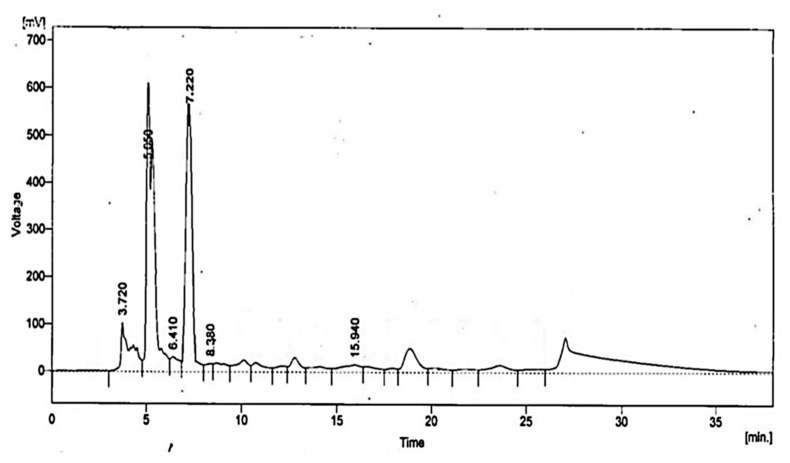
HPLC analysis of *L. coryniformis* BCH-4 AMCs for organic acids. The chromatogram has been labeled with peak retention time, i.e., 2-oxopropanoic acid (3.72 min), 2-hydroxypropane-1,2,3-tricarboxylic acid (5.05 min), 2-hydroxybutanedioic acid (6.41 min), 2-hydroxypropanoic acid (7.22 min), propanedioic acid (8.38 min) and butanedioic acid (15.94 min).

HPLC analysis of *L. coryniformis* BCH-4 AMCs revealed the presence of 2-oxopropanoic acid (Pyruvic acid), 2-hydroxypropane-1,2,3-tricarboxylic acid (Citric acid), 2-hydroxybutanedioic acid (Malic acid), 2-hydroxypropanoic acid (Lactic acid), propanedioic acid (Malonic acid) and butanedioic acid (Succinic acid) (Table 2; Figure 4).

**Figure 5:**
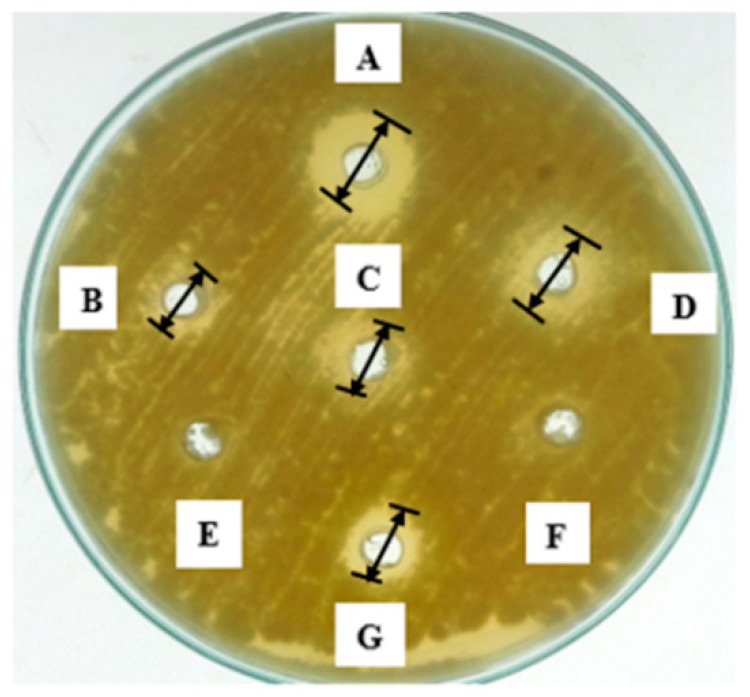
Antifungal potential of HPLC identified organic acids in *L. coryniformis* BCH-4 AMCs against *A. flavus*. A = Combination of acids, B = 2-hydroxybutanedioic acid, C = propanedioic acid, D = 2-hydroxypropane-1,2,3-tricarboxylic acid, E = 2-oxopropanoic acid F = butanedioic acid and G = 2-hydroxypropanoic acid. Well diameter was 8 mm.

The observed concentrations (g/L) were individually tested for antifungal potential against *A. flavus*. Individual organic acids have the zone of inhibition as; 2-hydroxypropanoic acid (5.33 ± 1.15 mm), 2-hydroxypropane-1,2,3-tricarboxylic acid (6.66 ± 0.57 mm), propanedioic acid (5.00 ± 1.00 mm), 2-hydroxybutanedioic acid (3.00 ± 1.00 mm), 2-oxopropanoic acid (~0.00 ± 0.00 mm) and butanedioic acid (~0.00 ± 0.00 mm), while the synergic effect of these organic acids exhibited a 9.66 ± 0.57 mm zone of inhibition (Figure 5).

### 2.7. Fourier Transform Infrared (FTIR) Spectroscopy Analysis

**Figure 6:**
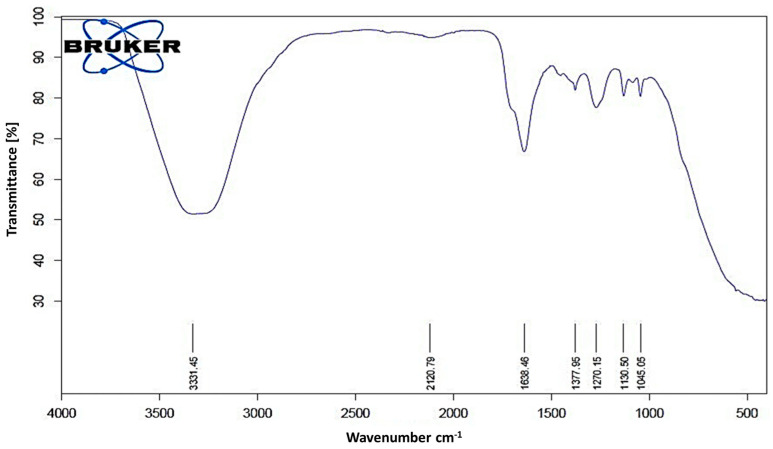
FTIR spectrum of *L. coryniformis* BCH-4 AMCs. The spectrum shows a range of 4000 to 500 cm^−1^ wave number (along x-axis) and % transmittance (along y-axis). The numbers above x-axis are representing specific functional group wave numbers (cm^−1^) which are present in *L. coryniformis* BCH-4 AMCs.

The FTIR spectroscopic analysis of *L. coryniformis* BCH-4 AMCs revealed the presence of several functional groups (Figure 6). The intense peak at 3331 cm^−1^ represents the characteristic hydroxyl (OH) group. The peak at 2120 cm^−1^ shows the presence of the C-C triple bond (alkyne group), while the peak at 1638 cm^−1^ indicates the presence of C=O stretching. The absorption peak at 1377 cm^−1^ shows the presence of alkane groups, while that at 1270 cm^−1^ shows C-O (acid) stretching. The peaks at 1130 and 1045 cm^−1^ indicate the presence of esters and alkyl halides. In the FTIR analysis spectrum, the presence of OH, C=O, C-O and esters functional groups indicated the characteristics of different organic acids, phenolic acids and fatty acids, that were responsible for the *L. coryniformis* BCH-4 AMCs bioactivities.

## 3. Discussion

*Aspergillus niger*, *A. fumigatus*, *A. flavus*, *Fusarium oxysporum* and *Rhizopus stolonifer* are the common pathogens of cereal grains in the field as well as during storage, that cause pre- and post-harvest spoilage in grains [11]. Chemical preservatives have been commonly used, although researches have shown concerns about their safety. Several studies have reported that these chemical preservatives may cause teratogenic, neurotoxic, and mutagenic effects [26]. Growth inhibition of pathogenic microorganisms by LAB and LAB AMCs looks to be a good biocontrol method [21] and suggests that they are good alternative to the chemical preservatives [27]. In the current study, in vitro bioactivities (antibacterial, antifungal and antioxidant) and the bioprotective potential of *L. coryniformis* BCH-4 AMCs to prevent the post-harvest loss in maize grains revealed promising results and it was observed that *L. coryniformis* BCH-4 AMCs had potent bioprotective potential against pathogens.

AMCs from *L. coryniformis* BCH-4 were extracted using ethyl acetate, a solvent that can extract a wide range of AMCs. The same solvent has been used for the extraction of AMCs produced by *Bacillus cereus* [28] and *L. plantarum* [29]. The findings of the current study show that *L. coryniformis* BCH-4 AMCs had potent in vitro antibacterial and antifungal activities (Figure 1) against tested pathogenic bacterial (*E. coli* and *S. aureus*) and fungal (*A. fumigatus* and *A. flavus*) strains. Inhibitory actions have also been reported for other *Lactobacillus* species, such as *L. bulgaricus*, *L. casei*, *L. plantarum*, *L. fermentum* against intestinal pathogens like *E. coli*, *S. aureus*, *Shigella dysenteriae* and *Salmonella paratyphi* A [30]. The AMCs of *L. plantarum* UM55 and *L. buchneri* UTAD104 also inhibited the growth of *Penicillium nordicum*, a foodborne pathogen [31]. Moreover, *L. coryniformis* BCH-4 AMCs also showed potent antioxidant activity (DPPH radical scavenging activity) in a concentration-dependent manner which is higher than reported earlier for other *Lactobacillus* species, such as *L. curvatus* SR6, which showed 59.67% ± 6.68% [32], while *L. plantarum* C88 exhibited 53.05% DPPH radical scavenging activity [33]. The DPPH scavenging activity assay was used because of its wide use, reliability, rapidness, simplicity and cost effectiveness as compared to other methods, e.g., ABTS. This assay is used to measure the ability of compounds to act as free radical scavengers or hydrogen donors, and to evaluate total antioxidant activity. It can also be used to quantify antioxidants in complex biological systems, for solid or liquid samples. It is a valid, accurate and highly reproducible method to evaluate radical scavenging activity of antioxidants, since the radical compound is stable and need not be generated. It also requires mild experimental conditions and is independent of sample polarity [34,35,36].

The wide spectrum of *L. coryniformis* BCH-4 AMCs has potential for several therapeutic applications. The remarkable antifungal activity of *L. coryniformis* AMCs against A. flavus (Figure 2A,B) make them a suitable and safe candidate for commercial applications [37] that can be used as good alternative to chemical preservatives [38,39]. The current study is the first report that describes the antifungal potential of *L. coryniformis* AMCs in comparison to FDA-approved food preservatives.

Moreover, *L. coryniformis* BCH-4 AMCs inhibited the growth of *A. flavus* on maize grains (Figure 3), while the proximate analysis (Table 1) showed that *L. coryniformis* BCH-4 AMCs significantly (*p* ≤ 0.05) improved the nutritional profile of treated maize grains. The increase in the nutritive value of maize is presumably due to addition of AMC extract that is a mixture of peptides, bacteriocins (protein in nature), exo-polysaccharides, fatty acids, organic acids, etc. [40,41]. The increase in moisture content of *A. flavus*-inoculated maize grains (A2) could be attributed to the hydrolysis of cellulose and pectin components of the cell wall by *A. flavus* as a possible mechanism of invasion and subsequent infection of maize tissue [42]. The crude fiber, fat, ash, protein and β-carotene contents were also significantly (*p* ≤ 0.05) decreased in A2 because protein, fiber and fat contents might have been broken down by pathogenic fungi into smaller molecules that are metabolized. Meanwhile, the ash content can be decreased in infected food due to the secretion of cell wall-degrading enzymes and by the toxin production of pathogens [43], while β-carotene can be broken down into β-ionone [44]. Complex molecules such as protein and polysaccharide are required by fungi to build up the hyphal wall and for obtaining energy by respiration [11,45]. Earlier reports describing the biopreservative property of *L. plantarum* AMCs against *A. flavus* used soybeans as a food model [39]. Khanafari et al. [46] also reported the bioprotective effects of *L. plantarum* PTCC 1058 against *A. flavus* in maize seeds. In that study, HPLC analysis clearly showed that *A. flavus* growth was 100% inhibited in *L. plantarum* inoculated maize seeds. In another study, 37 peptides identified in *L. plantarum* TE10 reduced the *A. flavus* growth on selected (GWG 111) maize seeds [47]. However, no study has been found that described the nutritional contents of cereals grains after the application of LAB compounds and/or the pathogenic effects of *A. flavus* on nutritional components. Meanwhile, the results of the current study clearly demonstrate that pathogens utilized the essential nutrients after infestation through their degradation activities [11,45].

HPLC analysis of *L. coryniformis* BCH-4 AMCs showed the presence of 2-oxopropanoic acid, 2-hydroxypropane-1,2,3-tricarboxylic acid, 2-hydroxybutanedioic acid, 2-hydroxypropanoic acid, propanedioic acid and butanedioic acid (Table 2). The individual and synergistic antifungal effect against *A. flavus* (Figure 5) showed the antimicrobial potential of organic acids. Organic acids such as lactic acid (2-hydroxypropanoic acid), citric acid (2-hydroxypropane-1,2,3-tricarboxylic acid), malic acid (2-hydroxybutanedioic acid), succinic acid (butanedioic acid) and malonic acid (propanedioic acid) are used as pH regulators, flavor enhancers, baking additives and biopreservatives in the food industry [48,49]. However, these results (synergic effect 9.66 ± 0.57 mm zone of inhibition) indicated that antifungal activity of *L. coryniformis* BCH-4 AMCs against *A. flavus* (12.33 ± 0.57 mm zone of inhibition) can also be contributed by other AMCs in addition to organic acids.

Moreover, the FTIR spectroscopic analysis (Figure 6) further validated the HPLC and antifungal results of *L. coryniformis* BCH-4 AMCs. The presence of hydroxyl (OH) 3331 cm^−1^ (3570–3200 cm^−1^) [50,51], alkyne 2120 cm^−1^ (2260–2100 cm^−1^), carbonyl 1638 cm^−1^ (1650–1600 cm^−1^) [51], alkane 1377 cm^−1^ (1480–1345 cm^−1^) [52], C-O 1270 cm^−1^ (1320–1210 cm^−1^) [51] and esters and alkyl halides groups 1130 and 1045 cm^−1^, respectively (1400–1000 cm^−1^) [52], demonstrated the characteristics of different organic acids [53] as well as other AMCs present in *L. coryniformis* BCH-4. The hydroxyl (OH) group’s presence in FTIR can be attributed to phenolic compounds which have already been reported in *Lactobacillus* species [54]. Different fatty acids are also present in LAB such as hydroxyisocapric acid, decanoic acid and 3-hydroxydecanoic acid isolated from *L. reuteri* ee1p that have great potential against dermatophytes [55]. Similarly, the hexadecenoic acid, 9,12-otadecadienoic acid (*Z*,*Z*)-methyl ester and methyl ester fatty acids have been found in *L. plantarum* and *L. coryniformis* [18]. The literature about *L. coryniformis* species is barely available, while the findings of the current study demonstrate the potential applications of *L. coryniformis* AMCs as safe natural alternatives to chemical preservatives for bioprotection of cereal grains against pathogen spoilage and to increase their nutritional values.

## 4. Materials and Methods

### 4.1. Chemicals, Reagents and Maize Grains

The de Man, Rogosa and Sharpe (MRS) agar/broth, glycerol (Analytical grade), nutrient agar/broth, Vogel’s agar/broth, ethyl acetate (HPLC grade), 2,2-diphenyl-1-picrylhydrazyl (DPPH), 2-oxopropanoic acid (pyruvic acid), 2-hydroxypropane-1,2,3-tricarboxylic acid (citric acid), 2-hydroxybutanedioic acid (malic acid), 2-hydroxypropanoic acid (malonic acid), propanedioic acid (lactic acid), butanedioic acid (succinic acid), β-carotene, sodium benzoate, potassium sorbate and sodium acetate were mainly purchased from Sigma-Aldrich, St. Louis, MO, USA. Maize grains (FMC Variety C-7065) were collected from a grain market in Faisalabad, Pakistan.

### 4.2. Microbial Cultures and Growth Media

*Lactobacillus coryniformis* BCH-4 (KX388387), previously isolated from fermented rice rinsed water with lactose as a carbon source [18] was cultured aerobically on MRS agar/broth at 37 °C for 48 h and preserved at −80 °C for long term storage in 15% (*v*/*v*) glycerol. Laboratory standard pathogenic strains were also available in the Industrial Biotechnology Lab, Department of Bioinformatics and Biotechnology, GCUF, Faisalabad, Pakistan. Tested bacterial strains *Escherichia coli* and *Staphylococcus aureus* were grown on nutrient medium at 37 °C for 16 h, while fungal strains *Aspergillus flavus* and *Aspergillus fumigatus* were grown on Vogel’s medium at 30 °C for 48 h before storage at 4 °C.

### 4.3. Extraction of Antimicrobial Compounds (AMCs)

*L. coryniformis* BCH-4 was cultured in 6L of MRS broth (pH 6.4 ± 0.2) at 37 °C for 72 h with constant stirring at 120 rpm in the fermenter (BioFer-010, ICCC, Islamabad, Pakistan). After 72 h, cell-free supernatant (CFS) of *L. coryniforims* BCH-4 was prepared by centrifugation (Z326K, Hermle, Wehingen, Germany) at 6000 rpm (4430× *g*) for 10 min at 4 °C and subsequently filtered through sterilized 0.22 μm pore-size filters (Advantec Toyo Kaisha, Ltd. Tokyo, Japan). AMCs were extracted from filter-sterilized CFS by the solvent phase extraction according to Wang et al. [56], with a few modifications. Ethyl acetate was used as an extracting solvent and AMCs were extracted by mixing same ratio of CFS and solvent [150 mL:150 mL] in an Erlenmeyer flask followed by shaking at 120 rpm on table-top shaker (Phoenix RS-OS 10, Irmeco, Lütjensee, Germany) for 2 h at room temperature. Mixture was poured in a separating funnel and allowed to stand until organic and aqueous phases were separated. The organic phase was collected, and the aqueous phase was further used to extract the maximum amount of AMCs in CFS. This process was repeated three times and organic phase was combined, concentrated by rotary evaporator (Rotavapor^®^ R-2100, Buchi, Flawil, Switzerland) under vacuum at lower than 40 °C. Ethyl acetate was evaporated and extracted dark brown concentrated (viscous) compounds were further evaluated for their bioactive potential.

### 4.4. Antimicrobial Activity

Antimicrobial activity assay was carried out by the agar well diffusion method [57] under aerobic conditions. Agar medium was prepared and poured into Petri dishes. Pathogenic bacterial strains (10^8^ CFU/mL) and fungal strains (10^6^ Spores/mL) as mentioned earlier were spread using sterile cotton swab over the plates and 8 mm diameter wells were punched into agar medium using a sterile cork borer. Then, 60 μL of extracted compounds was loaded into each well after diluting with distilled water (Water:AMCs 1:3). Non-inoculated MRS broth was used as a negative control. The plates were incubated for 16 h at 37 °C for antibacterial and for 48 h at 30 °C for antifungal activity. The antimicrobial potential was measured in millimeters (mm) as inhibition zones around wells using a ruler. The assay was performed in triplicate. Antimicrobial activity was determined by following the formula below [58]:

Diameter of zone of growth inhibition around the well (mm) − diameter of well (8 mm)

### 4.5. Antioxidant Activity

2,2-diphenyl-1-picrylhydrazyl (DPPH) scavenging activity was performed to evaluate the antioxidant potential of extracted compounds according to the method of Gangwar et al. [59], with a few modifications. One milliliter of 25, 50, 100 (μL/mL) concentrations of AMCs was mixed with 1 mL of DPPH ethanol (0.2 mM) solution and the reaction was left to stand at room temperature for 30 min. The absorbance of the sample (A_1_) was determined at 517 nm, while ethanolic DPPH solution (A_0_) was used as a control. The assay was performed in triplicate. The inhibition percentage (%) was calculated by the following equation:

DPPH radical scavenging age% = [(A_0_ − A_1_)/A_0_] × 100 (1)

where A_0_ is the absorbance of the DPPH solution and A_1_ is the absorbance of the sample.

### 4.6. Comparison of Antifungal Activity of AMCs with Commercial Preservatives

The antifungal potential of AMCs was compared with commercially available and FDA-approved preservatives. Sodium benzoate and potassium sorbate (dissolved in 20 mM sodium acetate buffer, pH 4) were used at the concentrations of 0.05%, 0.1%, 0.2% (*w*/*v*) and 0.01%, 0.05%, 0.1% (*w*/*v*), respectively (FDA-approved values). The antifungal activity was determined as described previously in the agar well diffusion method [57,58] at 30 °C after 48 h of incubation.

### 4.7. Bioprotection of Maize Using AMCs

The antimicrobial compounds (AMCs) produced by *L. coryniformis* BCH-4 were used for the bioprotection of maize according to the method developed by Yang and Chang [39], with minor modifications. After washing with distilled water, maize grains were soaked in extracted AMCs with different concentrations, prepared in sterilized distilled water [25%, 50%, 75%, and 100% (*v*/*v*) AMCs] for 8 h at room temperature. Six maize grains of each concentration were shifted to sterilized Petri plates. Besides this, maize grains soaked in MRS broth of different concentrations [25%, 50%, 75%, and 100% (*v*/*v*)] were used as a control. *A. flavus* spore suspension (10^6^ Spores/mL) was prepared and 12 μL of the suspension was spread on each maize grain in Petri plates. Inoculated maize grains were incubated at 30 °C and were examined after 48 and 72 h. The reason for selecting only *A. flavus* in this experiment is that it predominantly affects the maize grains [47].

### 4.8. Proximate Analysis

The proximate analysis was carried out at the Post Harvest Research Centre (Ayub Agricultural Research Institute, Faisalabad, Pakistan). The quality of untreated (raw) and treated maize grains was examined by calculating moisture, protein, fat, fiber and ash contents. Samples and solutions were prepared according to the methods of the Association of Officiating Analytical Chemists [60]. The untreated sample (raw maize grains) was labeled as A1, while the treated samples—*A. flavus* inoculated maize grains and *A. flavus* + AMCs inoculated maize grains—were labeled as A2 and A3, respectively.

### 4.9. Estimation of β-Carotene in Raw and Treated Maize

The concentration of β-carotene was determined according to Junpatiw et al. [61], with minor modifications. Freshly weighed 0.5 g of raw and treated maize grains were ground separately and mixed with 6 mL absolute ethanol containing 0.1% (*w*/*v*) butylated hydroxytoluene using a vortex mixer. The sample mixtures were placed in a water bath for 5 min at 85 °C. After that, 120 µL of (80% *w*/*v*) potassium hydroxide was added, vortexed and put in a water bath for 10 min at 85 °C to hydrolyze the carotenol ester present in sample mixtures. During saponification process, the samples were vortexed after every 5 min. In the samples, 3 mL of cold deionized water was added and then placed immediately in the ice bath. Furthermore, the samples were centrifuged at 3500 rpm (1400× *g*) for 5 min after adding and mixing 3 mL of petroleum ether and diethyl ether (2:1, *v*/*v*). The organic supernatant phase was collected after centrifugation and analyzed on HPLC (LC-10A, Shimadzu, Kyoto, Japan) equipped with YMC C30 5 μm, 4.6 × 250 mm carotenoid column (Waters, Milford, MA, USA). An isocratic mobile phase system consisting of acetonitrile:dichloromethane:methanol (70:20:10, *v*/*v*) was used with a flow rate of 1.0 mL/min and monitored at 450 nm with the help of UV-Vis detector (SPD-10A VP, Shimadzu, Kyoto, Japan). The carotenoid concentration in raw and treated maize grains was expressed as µg/100 g.

### 4.10. Determination of Organic Acids in AMCs Using High Performance Liquid Chromatography (HPLC)

For the analysis of organic acids, AMCs were mixed well by vortex, filtered through 0.22 μm syringe filter (Advantec Toyo Kaisha, Ltd. Tokyo, Japan) and injected in HPLC system (LC-10A, Shimadzu, Kyoto, Japan) fitted with Shim-Pack CLC-ODS (C-18), 5 μm, 25 cm × 4.6 mm column, Shimadzu LC-10AT pump and UV-Vis detector (SPD-10A VP, Shimadzu, Kyoto, Japan). The isocratic mobile phase (0.1% *v*/*v* H_3_PO_4_) with 0.5 mL/min flow rate at room temperature was used. The elute was observed at 215 nm by UV-Vis detector, data were collected and processed using the 32KARAT software operating system by default method for channel 2 of 2-oxopropanoic acid, 2-hydroxypropane-1,2,3-tricarboxylic acid, 2-hydroxybutanedioic acid, 2-hydroxypropanoic acid, propanedioic acid and butanedioic acid [18].

### 4.11. Antifungal Activity of Organic Acids

The determined concentrations of organic acids by HPLC were evaluated separately for antifungal potential against *Aspergillus flavus*. The antifungal activity of individual organic acids and their synergistic effect were determined using the plate method at 30 °C for 48 h as described previously [57,58].

### 4.12. Determination of Functional Groups by Fourier Transform Infrared (FTIR) Spectroscopy

FTIR spectroscopic analysis was done for the identification of specific functional groups [51]. The infrared absorption spectrum was recorded on the FTIR spectrophotometer (Tensor II, Bruker, Billerica, MA, USA) in the 4000 to 500 cm^−1^ region and spectrum was obtained as wave number versus percentage (%) transmittance.

### 4.13. Statistical Analysis

The zones of inhibition and antioxidant activity were calculated as the mean ± standard deviation of three replicates (n = 3). Meanwhile, one-way ANOVA (mean ± standard error) was performed for proximate analysis treatments (n = 3) using SPSS software package (Version 23.0, IBM Corporation, Armonk, NY, USA). Moreover, treatment means were separated by using Tukey’s HSD (Honest Significant Difference) at a significance level of 0.05. Differences at *p* ≤ 0.05 were considered significant.

## 5. Conclusions

The work represents a comprehensive analysis of the *L. coryniformis* BCH-4 antimicrobial compounds (AMCs) as antimicrobial, antioxidant and bioprotective agents of cereal grains. *L. coryniformis* AMCs were found to have strong inhibitory activity against food pathogens. The results highlight the higher nutritional value of AMC-treated maize grains than raw or *A. flavus*-infected maize grains. The detailed characterization of LAB (as starters, biocontrol agents, nutraceuticals and probiotics) can provide solid evidence-based support for their beneficial effects. It is also a promising approach to obtain functional foods and enhanced shelf life. In future, this work can be further extended to purify *L. coryniformis* AMCs for their potential use in the food industry and agriculture sectors.

## Figures and Tables

**Table 1:** Proximate analysis and β-carotene determination of un-treated and AMC-treated maize grains.

Parameters (%)	Treatments		
	A1 (Raw Grains)	A2 (*A. flavus* Inoculated Grains)	A3 (*A. flavus* + AMCs Inoculated Grains)
Moisture	23.90 ± 0.36 ^a^	26.26 ± 0.39 ^b^	24.00 ± 0.35 ^a^
Protein	8.63 ± 0.43 ^b^	6.30 ± 0.41 ^a^	10.73 ± 0.39 ^c^
Fat	3.26 ± 0.12 ^b^	1.96 ± 0.08 ^a^	3.90 ± 0.05 ^c^
Fiber	19.50 ± 0.30 ^b^	17.03 ± 0.32 ^a^	21.33 ± 0.12 ^c^
Ash	8.50 ± 0.40 ^b^	6.86 ± 0.31 ^a^	10.00 ± 0.11 ^c^
β-Carotene (μg/100 g)	241.33 ± 0.33 ^b^	235.66 ± 0.33 ^a^	242.00 ± 0.57 ^b^

Values are reported as mean ± standard error. Different letters in the same row show significant differences among each group, according to Tukey’s HSD test (*p* ≤ 0.05).

**Table 2:** Acid profile of *L. coryniformis* BCH-4 AMCs with their respective molecular weight, retention time and concentration.

Sr. No.	Identified Organic Acid	Molecular Weight (g/mole)	Retention Time (min)	Concentration (g/L)
1	2-Oxopropanoic acid	88.06	3.72	0.51
2	2-Hydroxypropane-1,2,3-tricarboxylic acid	192.12	5.05	11.87
3	2-Hydroxybutanedioic acid	134.08	6.41	0.79
4	2-Hydroxypropanoic acid	90.08	7.22	11.85
5	Propanedioic acid	104.06	8.38	0.003
6	Butanedioic acid	118.09	15.94	2.84

## Abbreviations

LAB	Lactic acid bacteria
CFS	Cell free supernatant
AMCs	Antimicrobial compounds
DPPH	2,2-Diphenyl-1-picrylhydrazyl
HPLC	High performance liquid chromatography
FTIR	Fourier transform infrared spectroscopy
